# Adult-onset leukoencephalopathy with vanishing white matter with compound heterozygous* EIF2B3* gene variants

**DOI:** 10.1186/s12883-024-03721-0

**Published:** 2024-06-13

**Authors:** Meilin Gui, Miao He, Lixia Qin

**Affiliations:** 1 Department of Neurology, The Second Xiangya Hospital, Central South University, 139# Renmin Road, Changsha, Hunan 410011 China; 2 National Clinical Research Center On Mental Disorders, Changsha, Hunan China

**Keywords:** Vanishing white matter, Adult-Onset, Leukoencephalopathy, Pathognomonic MRIs

## Abstract

**Background:**

Leukoencephalopathy with vanishing white matter (VWM) is an autosomal recessive disorder affecting the white matter of the brain. It typically manifests during childhood, with clinical features including sudden and severe neurological deterioration triggered by stressors such as febrile illness, minor head trauma, or stressful events. Adult-onset cases of VWM are exceptionally uncommon.

**Case presentation:**

In this case, we present an adult patient who exhibited late-onset progressive VWM characterized by ataxia, postural instability, cognitive impairment, and emotional disturbances. Comprehensive screening for endocrine, metabolic, tumor, and immunologic disorders yielded normal or negative results. Brain imaging revealed diffuse and confluent hyperintensity in the white matter on T2-weighted images, along with periventricular cavitations. Genetic testing confirmed the diagnosis of VWM, identifying two heterozygous variants in the eukaryotic translation initiation factor 2B subunit γ (*EIF2B3*) gene: a pathogenic variant, c.1037 T > C (p.I346T), and a variant of undetermined significance, c.22A > T (p.M8L). Upon a 2-year follow-up, the patient's symptoms deteriorated rapidly following a COVID-19 infection.

**Conclusions:**

In conclusion, we have presented a case of classical adult-onset VWM. Since there are no cures or definitive treatments for the disease, it's extremely important to focus on early diagnosis and the prevention of stressors to avoid acute deterioration.

## Background

Leukoencephalopathy with vanishing white matter (VWM) ranks among the most prevalent hereditary white matter disorders in children, though its exact incidence is still not clearly established [1, 2]. It holds the distinction of being the first known hereditary human disease linked to mutations in any of the five subunits of the eukaryotic translation initiation factor 2B (eIF2B α, β, γ, δ and ε) [3, 4]. Typically, VWM manifests in childhood with symptoms like cerebellar ataxia, moderate spasticity, vision loss, and mild seizures, while cognitive dysfunctions are usually less pronounced. However, there's a growing recognition of VWM in adults [5, 6]. Notably, adults with VWM often exhibit more significant cognitive and psychiatric issues compared to the primarily motor disabilities seen in early-onset cases. VWM generally follows a chronic and progressive course. Adults with the onset of VWM tend to experience a more favorable progression than those diagnosed in early childhood. However, both groups are prone to sudden neurological decline and unexplained comas, often triggered by stress factors like minor head injuries, feverish infections, or acute fright [7]. Additionally, female patients may face issues like premature ovarian failure and hyperprolactinemia.

Diagnosing VWM can be challenging due to its complex clinical manifestations, making it difficult to rely solely on clinical symptoms. However, distinctive magnetic resonance imaging (MRI) patterns, such as widespread and merging white matter lesions with periventricular cavitations, can offer crucial diagnostic hints [8]. A positive genetic test further confirms the diagnosis. In this report, we discuss a case of adult-onset VWM in a Chinese family, characterized by progressive gait impairment and dementia. This case was attributed to compound heterozygous variants in the *EIF2B3* gene.

## Case presentation

A 46-year-old woman came to our clinic with a four-year history of progressively worsening walking difficulties, erratic personality changes, and cognitive impairment. There were no significant family or personal medical histories, and no incidents of poisoning were reported. She has given birth to a daughter. Her symptoms started subtly and gradually worsened over time. Initially, she experienced weakness in her left extremities. There was a noticeable decline in her recent memory, counting ability, and orientation. Her family observed mood swings, with increased irritability and a short temper. She did not report any issues with blurred vision or double vision. Over time, her symptoms intensified. During the disease's progression, she developed urinary and fecal incontinence. About seven months ago, she fainted unexpectedly, leading to suspicions of head trauma. Following this incident, her neurological symptoms significantly deteriorated. Consequently, she was admitted to our hospital for further evaluation and treatment.

**Fig. 1:**
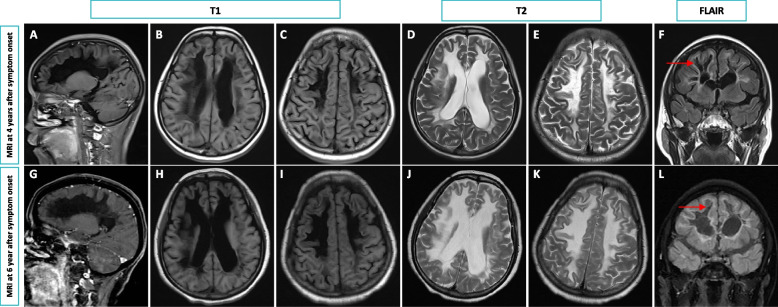
MRI changes of the patient at two time points. MRI obtained at 4 years (**A**-**F**) and 6 years (**G**-**L**) after the onset of the symptoms. Hypointense signal was observed on sagittal T1-weighted MRI (**A**, **G**). Axial MRI showed extensive, symmetric, confluent hyperintensities in the white matter, predominantly in the periventricular (**B**, **D**, **H**, **J**) and frontoparietal (**C**, **E**, **I**, **K**) regions, on the T1-weighted MRI (**B**, **C**, **H**, **I**) and hypointensities on the T2-weighted MRI (**D**, **E**, **J**, **K**). Coronal FLAIR images (**F** and **L**) revealed the affected white matter adjacent to the anterior and posterior horns of lateral ventricles had a signal intensity identical to that of CSF, which was suggestive of cystic degeneration (Red arrow)

**Fig. 2:**
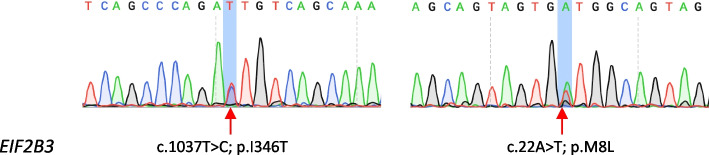
Genetic results of the patient. Sanger sequencing confirmed heterozygous variants in the *EIF2B3* gene: c.1037 T > C (p.I346T) and c.22A > T (p.M8L)

A neurological examination of the patient revealed cerebellar ataxia, postural instability, and exaggerated tendon reflexes. She was unable to perform a tandem gait. On the Mini-Mental State Examination, she scored 15 out of 30, indicating significant impairments in time orientation, counting ability, and delayed recall. An extensive range of tests was conducted. Routine lab tests of blood, urine, and cerebrospinal fluid (CSF), including lactate and pyruvate levels, showed no abnormalities. Screening for endocrine, metabolic, and immunologic disorders, as well as tumor markers, returned normal or negative results. Ovarian function was not assessed due to fertility issues and financial constraints, making it impossible to determine ovarian failure. Brain MRI scans revealed diffuse, confluent white matter hyperintensities in the frontoparietal and periventricular regions, and the cerebellum on axial T2-weighted images. Coronal fluid-attenuated inversion recovery (FLAIR) sequences showed periventricular cavitations (Fig. 1). Electrophysiological testing of motor and sensory nerve conduction velocities revealed no abnormalities, and spinal MRI results were unremarkable. The brain MRI findings strongly suggested VWM, prompting genetic testing. Whole-exome sequencing (WES) identified heterozygous variants in the *EIF2B3* gene: a known pathogenic variant c.1037 T > C (p.I346T), and a variant of uncertain significance, c.22A > T (p.M8L). These were further confirmed by Sanger sequencing (Fig. 2). Gene testing for her parents was not available because they had passed away. *EIF2B3* c.22A > T (p.Met8Leu) results in a conservative amino acid change in the Nucleotidyl transferase domain of the encoded protein. The variant allele was found at a frequency of 0.001427 within the East Asian subpopulation in the gnomAD database. In silico predictions for the variant in *EIF2B3* show it is tolerable according to SIFT, benign according to PolyPhen-2, disease-causing according to MutationTaster, tolerable according to PROVEAN, tolerable according to REVEL, and damaging according to CADD. ClinVar has classified the variant as of uncertain significance. Based on the distinctive MRI and genetic results, a diagnosis of VWM was made. The patient was treated with vitamin B and symptomatic care, which stabilized her symptoms for a considerable period. However, at the 2-year follow-up, she exhibited clinical and radiographic deterioration (Fig. 1), which was exacerbated by a COVID-19 infection.

## Discussion and conclusion

Leukoencephalopathy with VWM is an autosomal recessive disorder affecting white matter. The disease typically presents as a chronic and progressive condition, punctuated by episodes of rapid deterioration following feverish infections or head trauma [3]. While VWM is probably the most common form of genetic leukoencephalopathy in children, it has become increasingly recognized in adults in recent years [5, 6].

The underlying pathophysiology of the disease remains poorly understood. VWM is caused by mutations in any of the genes that encode the five subunits of eIF2B, ranging from *EIF2B1* to *EIF2B5*. eIF2B, a protein complex ubiquitously expressed in the body, is critical in regulating protein synthesis rates. Mutations in any *eIF2B* variants can impair protein synthesis inhibition and amplify the stress response, potentially explaining the acute neurological deterioration seen after minor head injuries or feverish infections [2]. Mutations in the *EIF2B5* gene are the most prevalent, representing about 56% to 70% of all VWM cases [6, 9, 10]. Additionally, a significant number of adult-onset cases have been linked to mutations in the *EIF2B5* gene [11]. Mutations in *eIF2B3* account for 4% of the mutations reported in patients of European descent, while they represent approximately 29% in the Chinese population [6]. Moreover, compared to patients of European descent, the incidence of epilepsy in Chinese adult-onset VWM patients is lower, but the incidence of optic neuropathy is higher [6]. Nevertheless, it is important to note that the current sample size is relatively small, and larger studies are needed in the future to identify any potential ethnic-specific differences. In addition, the relationship between specific genotypes and clinical phenotypes largely remains largely unknown. Previous studies have observed intra-familial phenotypic variability, indicating that environmental or epigenetic factors might significantly contribute to this heterogeneity [12]. Future reports on individual cases and case series will be invaluable in more accurately delineating the connection between genotype and phenotype.

Limited longitudinal studies suggest that the age of onset is a critical prognostic factor, with the severity of VWM generally being inversely related to the age of onset [13]. Adults with VWM often experience motor disturbances, cognitive decline, autonomic dysfunctions, visual problems, seizures, and behavioral changes [5]. Notably, adult-onset VWM tends to involve milder symptoms and a slower decline in neurological function, with more pronounced cognitive impairments compared to the predominantly motor disabilities seen in early-onset cases. Additionally, seizures and episodic exacerbations are significant factors in disease progression for patients of all ages, highlighting the importance of effective seizure management and preventative measures such as infection control, head injury prevention, and emotional stability. Signs of ovarian failure, including primary or secondary amenorrhea, irregular menses, or infertility, were indeed reported in most of female patients. Therefore, ovarian dysfunction is an important clue in diagnosing VWM. The patient in this report, presenting with classic clinical features like ataxia, cognitive impairment, emotional disturbance, and neurological decline, followed a chronic progressive course with acute neurological deteriorations post head trauma and COVID-19 infection.

In almost all cases, brain MRI reveals white matter rarefaction in VWM patients [8]. Cystic degeneration within diffuse white matter abnormalities is a highly sensitive and specific indicator of the disease. In end-stage VWM patients, the white matter vanishes, and large cystic foci typically develop in the subparietal regions. Although VWM is a rare neurological condition, its diagnosis is relatively straightforward when characteristic MRI patterns are present. Thus, it's crucial for physicians to recognize these distinctive MRI features. However, diagnosing adult-onset VWM can be more challenging. In these patients, white matter demyelination may progress slowly, often initially presenting as atrophy without cystic degeneration for several years. The presence of even minimal cavitations in the frontal periventricular white matter is a key indicator for diagnosis. When the disease is suspected, radiologists should specifically look for this finding, as it can prompt the diagnosis. The differential diagnosis for leukoencephalopathy is extensive, encompassing conditions such as mitochondrial leukoencephalopathies, adrenoleukodystrophy, metachromatic leukodystrophy, and hereditary diffuse leukoencephalopathy with neuroaxonal spheroids (HDLS), among others [7]. Genetic testing plays a vital role in confirming the final diagnosis. Currently, there are no cures or definitive treatments for VWM. It is strongly recommended to prevent cranial-cerebral injuries, febrile infections, and emotional instability as measures to avoid acute deterioration.

In conclusion, we present a case of adult-onset VWM with compound heterozygous variations in *EIF2B3*. It's important to consider VWM in adult patients who exhibit early-onset dementia and extensive white matter changes on MRIs. The most important lessons we can learned in this case maybe early detection of this condition can enable the implementation of preventive measures which are crucial in reducing episodes of rapid decline triggered by stress, potentially slowing the progression of the disease. In the future, more comprehensive case series and longitudinal studies are necessary to broaden our understanding of VWM.

## Data Availability

The datasets generated during and/or analyzed during the current study are available from the corresponding author on reasonable request.

## Abbreviations

VWM: Leukoencephalopathy with vanishing white matter
EIF2B3: Eukaryotic translation initiation factor 2B subunit γ
eIF2B: Eukaryotic translation initiation factor 2B
MRI: Magnetic resonance imaging
CSF: Cerebrospinal fluid
FLAIR: Fluid-attenuated inversion recovery
WES: Whole-exome sequencing
HDLS: Hereditary diffuse leukoencephalopathy with neuroaxonal spheroids
